# An exception to the matched filter hypothesis: A mismatch of male call frequency and female best hearing frequency in a torrent frog

**DOI:** 10.1002/ece3.2621

**Published:** 2016-12-20

**Authors:** Longhui Zhao, Jichao Wang, Yue Yang, Bicheng Zhu, Steven E. Brauth, Yezhong Tang, Jianguo Cui

**Affiliations:** ^1^ Chengdu Institute of BiologyChinese Academy of SciencesChengduSichuanChina; ^2^ Ministry of Education Key Laboratory for Tropical Plant and Animal EcologyCollege of Life SciencesHainan Normal UniversityHaikouHainanChina; ^3^ Department of PsychologyUniversity of MarylandCollege ParkMDUSA

**Keywords:** acoustic structure, *Amolops torrentis*, auditory brainstem response, auditory sensitivity, matched filter hypothesis, stream noise

## Abstract

The matched filter hypothesis proposes that the tuning of auditory sensitivity and the spectral character of calls will match in order to maximize auditory processing efficiency during courtship. In this study, we analyzed the acoustic structure of male calls and both male and female hearing sensitivities in the little torrent frog (*Amolops torrentis*), an anuran species who transmits acoustic signals across streams. The results were in striking contradiction to the matched filter hypothesis. Auditory brainstem response results showed that the best hearing range was 1.6–2 kHz consistent with the best sensitive frequency of most terrestrial lentic taxa, yet completely mismatched with the dominant frequency of conspecific calls (4.3 kHz). Moreover, phonotaxis tests show that females strongly prefer high‐frequency (4.3 kHz) over low‐frequency calls (1.6 kHz) regardless of ambient noise levels, although peripheral auditory sensitivity is highest in the 1.6–2 kHz range. These results are consistent with the idea that *A. torrentis* evolved from nonstreamside species and that high‐frequency calls evolved under the pressure of stream noise. Our results also suggest that female preferences based on central auditory system characteristics may evolve independently of peripheral auditory system sensitivity in order to maximize communication effectiveness in noisy environments.

## 1 Introduction

Animal communication involves transmission of complex signals from senders to receivers. In insects, anurans, birds, and mammals, acoustic signals play an important role in coordinating reproductive behavior (Gerhardt & Huber, 2002; Rogers & Kaplan, 2000). A minimum requirement of any successful animal communication system is that it provides unambiguous information about species identity and the sexual characteristics of the signaling individuals (Endler, 1993; Ryan & Rand, 1993). For example, in acoustic communication systems, auditory tuning generally tends to evolve toward improving the detection of biologically relevant acoustic signals in the natural environment. This reduces the probability of interactions occurring which can reduce the fitness of reproductive individuals such as hybridization and competition for a communication channel (Pfennig & Pfennig, 2009; Ritchie, 2007; Ryan & Rand, 1993). For this reason, the matched filter hypothesis predicts that the tuning of receivers’ auditory sensitivity will evolve to closely match the dominant frequency (DF) of species‐specific advertisement calls and the spectral energy distribution of male acoustic signals (Capranica & Moffat, 1983).

Nevertheless, vocal communication is often constrained by biotic and abiotic sources of environmental noise. Noise sources of biotic origin are mainly those of conspecific and heterospecific calls; sources of abiotic noise mainly include wind, rain, running water, and various anthropogenic activities, such as those associated with industry, traffic, and transportation (Barbosa & Cardoso, 2005; Parris, Velik‐Lord, & North, 2009; Penna, Pottstock, & Velasquez, 2005). There is clear evidence that conspecific choruses and continuous broadband noise decrease both the detection and discrimination of conspecific signals (Brumm & Slabbekoorn, 2005; Schwartz & Gerhardt, 1989; Wollerman, 1999), which in some cases may reduce reproductive fitness in both sexes. For instance, animals make errors when relevant signals are masked by high levels of background noise or when relevant and irrelevant signals are similar (Wollerman & Wiley, 2002). Such errors can lead to hybridization if individuals respond to the signals of closely related species (de Kort, den Hartog, & ten Cate, 2002).

Streamside breeding species have been reported to communicate by means of signals detuned from the noise spectra; for instance, the concave‐eared torrent frog (*Odorrana tormota*), the large odorous frog (*Odorrana graminea*), and the songbird (*Abroscopus albogularis*) produce calls containing ultrasonic components to avoid masking by the wideband background noise (Feng et al., 2006; Narins et al., 2004; Shen, Xu, Feng, & Narins, 2011; Shen et al., 2011). On the other hand, many morphological and physiological features of the anuran auditory system are conserved in phylogeny having changed little during the course of evolution (Wilczynski, Rand, & Ryan, 2001). Therefore, a mismatched relationship might arise between the acoustics of the sender's signals and the receiver's auditory sensitivity because these might change at different rates under the influences of natural and sexual selection (Gerhardt, 1994; Ryan & Brenowitz, 1985; Ryan, Perrill, & Wilczynski, 1992; Wilczynski et al., 2001). For species living near noisy streams, male calls might change more quickly than the sensitivity of the auditory system of females resulting in a mismatch between the spectral characteristics of male calls and female auditory sensitivity (Mason, Morris, & Hoy, 1999).

**Figure 1:**
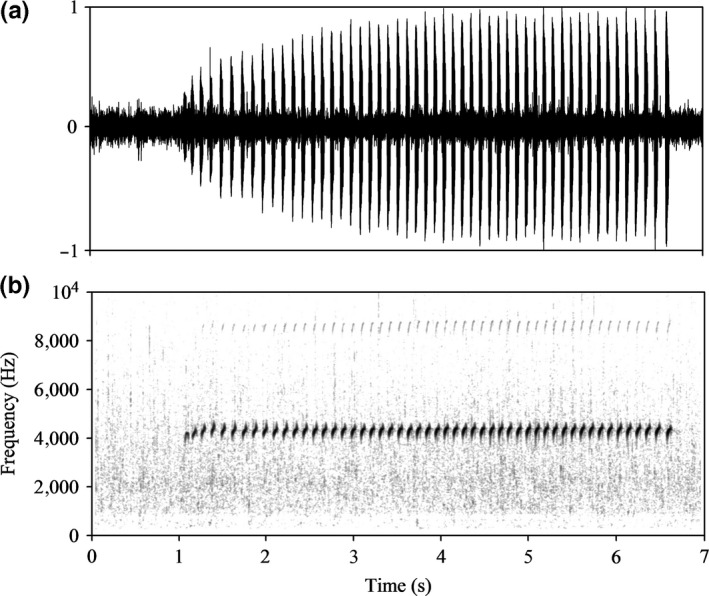
Acoustic characteristics of the natural advertisement call of *Amolops torrentis* (*A. torrentis*) and the streamside acoustic environment. (a) Waveform of a representative advertisement call with 52 notes. (b) Spectrograms of the recording showing the energy contained in the ambient noise. The increased energy at 4–5  kHz represents the advertisement call of *A. torrentis*. The background noise (significant energy below 4 kHz) is due to the fast‐flowing stream

The little torrent frog, *Amolops torrentis*, inhabits the rocks of mountain streams or near vegetation in Hainan Island, China. During the breeding season, males of this species produce calls consisting of a series of identical repeated notes (Figure 1) throughout the day and night. In this study, we first recorded and analyzed the acoustic features of male advertisement calls and that of the environmental background noise. Second, we measured the auditory brainstem response (ABR) of males and females in the laboratory in order to examine the relationship between auditory tuning curves and the spectral characteristics of male calls. Sexual dimorphism in auditory sensitivity may result from differences in the mechanical characteristics of the ear such as sexually dimorphic middle ears and tympanic membranes (Hetherington, 1994; Shen, Xu et al., 2011; Shen et al., 2011). There is obvious sexual size dimorphism in *A. torrentis*, so we also compared the auditory tuning curves of males and females.

The ABR method is a minimally invasive technique which has proven to be highly valuable in performing studies of auditory sensitivity in natural populations (Schrode, Buerkle, Brittan‐Powell, & Bee, 2014; Zhang, Cui, & Tang, 2012). Schrode et al. (2014) have verified that this is an effective method to study audition in anurans by comparing ABR audiograms with invasive multiunit recordings from the auditory midbrain. A few studies have also recorded auditory evoked potentials using less invasive subdermal procedures. Notably, the ABR technique has been shown to be sensitive enough to detect high‐frequency neural activity associated with activation of the basilar papilla (BP) (Katbamna, Langerveld, & Ide, 2006; Schrode et al., 2014). Nevertheless, this technique is not adequate for assessing female call preferences in different acoustic contexts such as differences in background noise.

Persistently high‐level background noise produced by rapidly flowing water is a powerful selective force causing adaptive evolution of acoustic signals and auditory systems for anuran species inhabiting areas alongside streams (Feng et al., 2006; Narins et al., 2004). Furthermore, stream noise is quite complex in rapid torrential environments and thus may act on intersexual selection. Pertinent to this, female phonotaxic behavior is a useful method for evaluating female preferences and can thus be used as a proxy for evaluating how intersexual selection may have acted on male calls. For these reasons, we also compared female phonotaxic responses to call playbacks with the DF of the calls adjusted to that of the best hearing sensitivity (which was relatively low) versus calls whose DF was adjusted to the natural call frequency of males (which was relatively high) in the presence of three levels of stream noise as described below, in order to determine whether the female behavioral response was influenced by the background noise context.

## 2 Materials and Methods

### 2.1 Study site and subjects

The study site was located in the Mt. Diaoluo Nature Reserve (18.44°N and 109.52°E), Hainan Province, China. Frogs were collected (between 0900 and 2200 hr) during the reproductive season, from April to August between 2014 and 2015. Daily temperatures varied between 14 and 25°C in this period. Frogs were brought to field research bases at Mt. Diaoluo, placed in containers with water and stones from their capture sites, and maintained at environmental humidity levels. Almost all individuals completed the entire test on the same day and were then returned in their containers to their collection sites where they were released. All phonotaxis and ABR recordings were completed within 48 hr after the animals were captured, and the frogs were returned to their natural habitat immediately after body size was determined. The subjects (13 males and 32 females) tested in this study had a body mass between 2.5 and 6.1 g (Mean ± *SD*; females: 5.2 ± 0.55 g; males: 2.7 ± 0.18 g) and a snout‐urostyle length between 27.5 and 42.0 mm (Mean ± *SD*; females: 39.0 ± 1.3 mm; males: 32.3 ± 3.6 mm). The number of individuals used in the field recordings, ABR measurements, and phonotaxis tests were 18, 23, and 39, respectively. ABRs were measured between 1300 and 1700 hr, and temperature during the experimental period ranged from 22 to 25°C. Prior to releasing each animal, we performed toe‐clip operations to insure each was not recorded and tested again. The frogs were used for the experiments with the permission of the management office of the Mt. Diaoluo Nature Reserve. All animal procedures were approved by the Animal Care and Use Committee of the Chengdu Institute of Biology, CAS.

### 2.2 Sound recordings and analysis

Sound pressure levels (dB re 20 μPa) were measured (A‐weighted) with a sound level meter (AWA 6291; Hangzhou Aihua Instruments Co.), and vocalizations were recorded with a directional microphone (Sennheiser ME66 with K6 power module) connected to a digital recorder (Marantz PMD 660, 16 bit, 44.1 kHz). The A‐weighted measurements exhibit significant attenuation below ~600 Hz; thus, there may be more noise at low frequencies than presented. Once a vocalizing male was located, the microphone connected to the recorder and the microphone connected to the sound level meter were fastened together to record advertisement calls and sound pressure levels, respectively, 1 m from the subject. As sound radiation varies in its directionality, the microphone and sound level meter were directed toward the snout‐vent orientation of the subject. Six to 10 calls were recorded continuously during each recording session for each male, and the peak sound pressure value was recorded for each call. After the recordings were completed, the frogs were captured and ambient SPLs (sound pressure levels) were measured at the location of their heads.

The waveform and spectrogram of male calls with background noise were prepared using PRAAT software (Boersma and Weeninkk, Version 5.1.11, University of Amsterdam). Calls were analyzed using Adobe Audition 3.0 software (CA, USA). Seven call properties were measured to quantify the characteristics of advertisement calls, including the fundamental frequency, maximum frequency, DF, call durations, notes per call, rising notes per call (the number of notes from the call onset to the note of largest amplitude), note duration, and internote interval. Frequency data were obtained from power spectra generated by fast Fourier transformation in the middle of the note (window type: Blackman–Harris; transform size: 1,024 points). To accurately measure the call amplitude, we subtracted the background noise from that of the signal using this formula: $L_{\mathrm{sig}}=10\;{\text{log}}_{10}\left(10^{\left(L_{\mathrm{sig}+\mathrm{noise}}/10\right)}-10^{\left(L_{\mathrm{noise}}/10\right)}\right)$, where *L*_sig + noise_ is the total sound pressure level, *L*_noise_ is the background noise level alone, and *L*_sig_ is the SPL of the signal (Brumm & Zollinger, 2011). Average values of call amplitude were calculated after separation from the total sound pressure level.

### 2.3 ABR measurements

All ABR measurements were conducted inside a soundproofed mini‐acoustical chamber (dimensions: 0.5 × 0.5 × 0.5 m) with walls and ceiling covered with acoustic foam to reduce reverberations. All animals were anesthetized lightly with a 0.2% MS‐222 (tricaine methane sulfonate) solution to a level at which the animals no longer responded to a toe‐pinch (~2–4 min), in order to standardize the procedure so that level of anesthesia would not bias the ABR recordings. Frogs were placed in a natural posture facing a speaker (SME‐AFS, Saul Mineroff Electronics, Elmont, NY, USA) that was used for broadcasting sound. Then, three 27‐gauge subdermal needle electrodes (Rochester Electro‐Medical, Inc., FL, USA) were inserted just under the skin at the vertex, above the tympanum and in the contralateral front leg, respectively. The stimulus presentation, ABR acquisition, equipment control, and data management were similar to a previous study in the Emei music frog (Zhang et al., 2012).

Briefly, stimulus generation and ABR recordings were carried out using a digital processor RM2 (Tucker‐Davis Technologies, Gainesville, USA) linked via fiber optic cables linked via RA4 cables and a USB linked to a computer running the custom software QuickABR. Two types of stimuli, tone pips and clicks, were delivered through a portable amplified field speaker (SME‐AFS; Saul Mineroff Electronic Inc, USA) which was placed 10 cm in front of the frog's head. Tone pips were used to measure auditory thresholds for each frequency at each intensity level, while broadband clicks were used to verify the presence of a biological signal in response to sound at each intensity level (as a control). Before ABR recordings, sound stimuli, from 0.8 to 18 kHz, were calibrated using a G.R.A.S. 46 BE 1**/**4 inch microphone (G.R.A.S. Sound & Vibration, Denmark) at the approximate position of the frog's head. Tone bursts were synthesized digitally from 0.8 to 18 kHz (0.8, 1.0, 1.2, 1.6, 1.8, 2.0, 2.4, 2.8, 3, 3.5–6 [with 0.5 kHz steps], 6–10 [with 1.0 kHz steps], 10–18 [with 2.0 kHz steps]), with a stimulation duration of 1 ms rise**/**fall time and 3 ms plateau time and sample rate of 24,414 Hz. At each frequency, we recorded ABRs at nine intensity levels ranging from 90**/**85 to 45**/**40 dB in 5 dB SPL steps. For all stimuli, we obtained two replicate averages of the ABR, each based on averaging responses to 400 stimulus repetitions. All biological signals were notch filtered at 50 Hz during data collection.

The ABR thresholds were determined with methods similar to that described by Brittan‐Powell, Christensen‐Dalsgaard, Tang, Carr, and Dooling (2010). Threshold measurements were defined as the lowest stimulus level for which no repeatable responses could be recognized by visual detection. For each stimulus, we compared two replicate averages in which the lowest threshold was collected.

### 2.4 Female phonotaxis experiments

**Figure 2:**
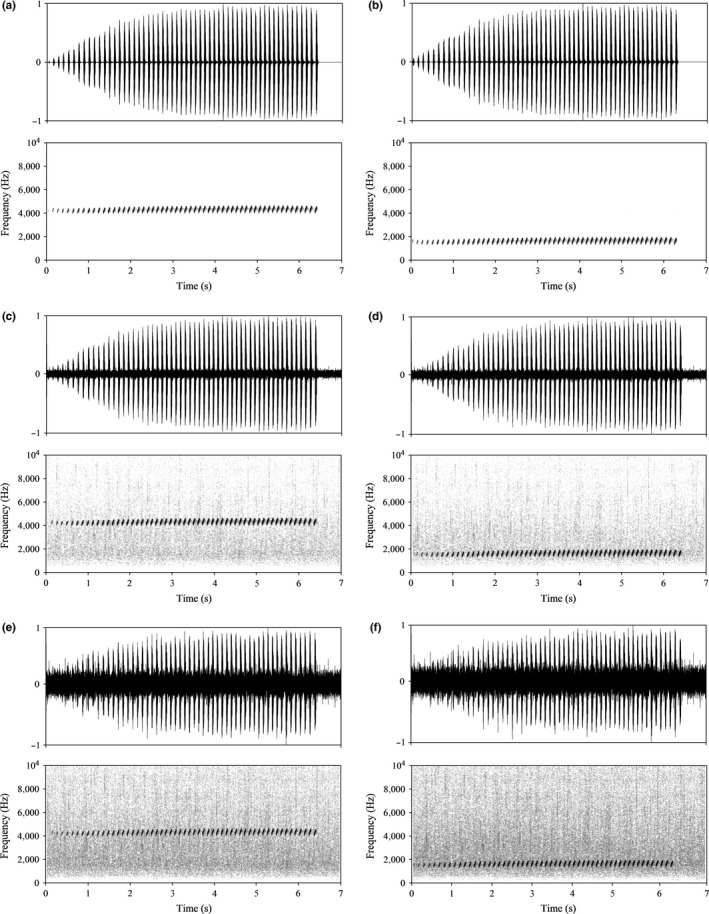
Waveforms (top) and spectrograms (bottom) of exemplars of the six types of call stimuli used in the female phonotaxis experiments. a (b) ‐ high (low) dominant frequency call with no noise added, c (d) ‐ high (low) dominant frequency call with low amplitude noise added, e (f) ‐ high (low) dominant frequency call with high amplitude noise added

Persistent background noise occurs in the habitat of the little torrent frog (55.6–79.5 dB). Thus, we performed two‐speaker phonotaxis tests offering females a choice between synthesized low DF calls (i.e., at the most sensitive female frequency as described in Section “3”) and high DF calls (i.e., at the natural male call frequency) with no noise added, a low noise level added (62 dB) or a high noise level added (74 dB), respectively. Additionally, we also tested whether the low‐frequency call is attractive to females compared to white noise. All acoustic stimuli (Figure 2) were synthesized using Avisoft SAS‐Lab Pro (Avisoft Bioacoustics, Berlin) and Adobe Audition 3.0 software, based on parameters derived from 13 different calling males and the background noise at their recording sites. The DF of synthetic calls was 4,318 Hz (high frequency) and 1,600 Hz (low frequency). These DFs correspond to those of natural male calls (Figure 1) and to that of the most sensitive frequency range of females, as described below in the Results section. The temporal characteristics of the signals remained unchanged. The background noise was synthesized from recordings obtained from different locations because the noise spectra can vary and then adjusted so that the amplitude would yield the desired signal to noise ratio. Synthesized calls and noise were combined to produce each stimulus.

We conducted the phonotaxis experiments in a sound‐attenuating chamber [2.2 (L) × 1.5 (W) m]. The female's behavior was observed on a monitor using a wide‐angle lens video system with an infrared light source. We placed each female in the center of the chamber, while the stimulus pairs were broadcast antiphonally from speakers (SME‐AFS; Saul Mineroff Electronics, Elmont, NY, USA) in the center of the walls opposite to one another such that the peak amplitude of each test call at the center of the arena was 80 dB SPL (re 20 μPa). Stimuli were presented with 5‐s interstimulus intervals. A positive response was scored if females approached the speaker within 10 cm without simply following the wall. A female was scored as not exhibiting phonotaxis if she was motionless for the first 5 min or for any subsequent two minutes of the trial, or spent more than 10 min roaming the arena without approaching a speaker. To control for potential side biases, we randomized the speaker assignments for each stimulus pair. The phonotaxis results showed that there were no side biases.

### 2.5 Statistical analysis

The normality of the distribution and homogeneity of variance of the values were tested using the Shapiro–Wilk and Levene tests, respectively. The DF of advertisement calls and the main energy distribution of stream noise were not normally distributed. The analysis between the DF of calls and the DF of stream noise was completed with the nonparametric Wilcoxon signed‐rank test. The comparison between the DF of the calls and the frequency of greatest sensitivity was made using the nonparametric Mann–Whitney rank sum test. The Kruskal–Wallis one‐way analysis of variance on ranks was employed to evaluate differences in the SPLs of calls and noise because these data are not independent.

Cross‐correlation analyses were performed: (1) to compare female audiograms and the spectra of vocalizations and (2) to compare male and female audiograms (Moreno‐Gómez, Sueur, Soto‐Gamboa, & Penna, 2013). Samples of male calls, male ABRs, and female ABRs were averaged, and the resulting average audiogram and average spectra were subjected to cross‐correlation analysis (Moreno‐Gómez et al., 2013). The cross‐correlation *r* was computed for all delays, thus resulting in a cross‐correlation series of twice the length as the original series. The 95% confidence interval was estimated by obtaining the 0.025 and 0.975 quantiles of the statistical distribution (Crawley, 2007). Repeated measures analysis of variance (ANOVA) was used to evaluate the effects of frequency and sex on tone‐evoked responses. Because the best hearing sensitivity was in the 1–3 kHz band and the spectral energy distribution of male acoustic signals was in the 3–6 kHz band (see Section “3”), cross‐correlation coefficients and ANOVA were obtained at 0.8–2.8 kHz, 2.8–6 kHz, and 7–18 kHz, respectively. Fisher's exact test was used to evaluate the phonotaxis data. All data were statistically analyzed with the SigmaPlot 11 software program (Systat Software Inc., San Jose, USA) and SPSS 16.0 software (SPSS Inc., USA). A significance level of *p *<* *.05 was used in all comparisons.

## 3 Results

### 3.1 Call characteristics and ambient noise

**Figure 3:**
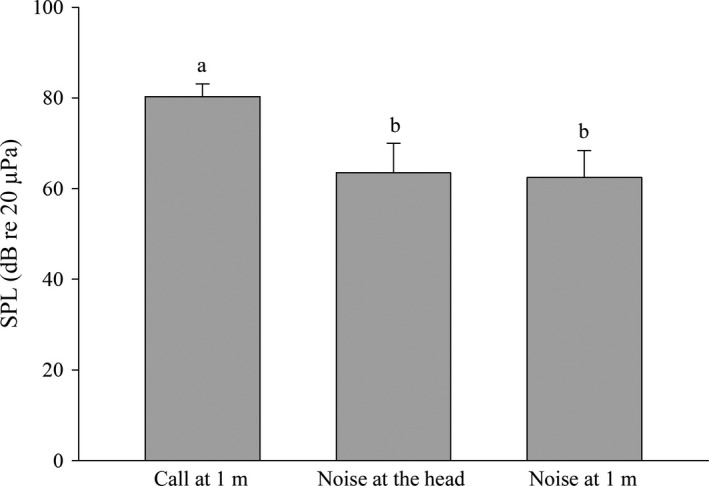
Mean sound pressure levels (+*SD*) of advertisement calls at 1 m from the subject, stream noise recorded at the position of the frogs’ head and stream noise recorded 1 m from the frog (*n* = 18). Values which do not share a common superscript letter differ significantly at *p* < .05

As shown in Table 1, the advertisement calls of *A. torrentis* exhibit an average duration of 6.43 ± 1.03 s and were comprised of 57 ± 13 short notes with an internote interval of 70 ± 9 ms. The relative call amplitude increased during the first 32 ± 8 notes, while average note durations (range 44–50 ms) varied somewhat. The noise of running water from nearby creeks exhibited significant energy in the 0–4 kHz range, which overlaps the fundamental frequency of the advertisement calls (range 3,604–3,820 Hz) (Table 1; Figure 1). However, the mean call DF was 4,318 Hz (range 4,134–4,565 Hz), which is statistically significantly different than the upper limit of the main energy distribution of stream noise (*n* = 11; Wilcoxon signed‐rank test: *Z* = −2.944, *p *<* *.001) although the frequency difference is only 300 Hz. The peak sound pressure of calls was 80.3 ± 2.8 dB at a distance of 1 m. The average sound pressure of the background noise was 62.4 ± 6.0 dB and 63.5 ± 6.5 dB, respectively, at the call recording sites 1 m from the location of the male subjects and at the position of the frogs’ head. Kruskal–Wallis one‐way analysis of variance on ranks indicated that the sound pressure between advertisement calls and noise differed significantly (*n* = 18, *q*_1_ = 6.884, *p*_1_ < .05, average background vs. calls at 1 m from the subject; *n* = 18, *q*_2_ = 7.364, *p*_2_ < 0.05, background and calls at 1 m from the subject), while the sound pressure of ambient noise was not significantly different at the two measurement sites (*n* = 18, *q* = 0.479, *p *>* *.05) (Figure 3).

**Table 1:** Means, standard deviations, and maximum and minimum values of call parameters

Call parameter	Mean	*SD*	Max	Min
Fundamental frequency (Hz)	3,696	41	3,820	3,604
Maximum frequency (Hz)	4,528	65	4,617	4,278
Dominant frequency (Hz)	4,318	167	4,565	4,134
Call duration (s)	6.43	1.03	7.60	4.78
Notes per call	57	13	76	40
Rise notes per call	32	8	48	24
Note duration (ms)	46	2	50	44
Internote interval (ms)	70	9	81	52

### 3.2 ABR recordings

**Figure 4:**
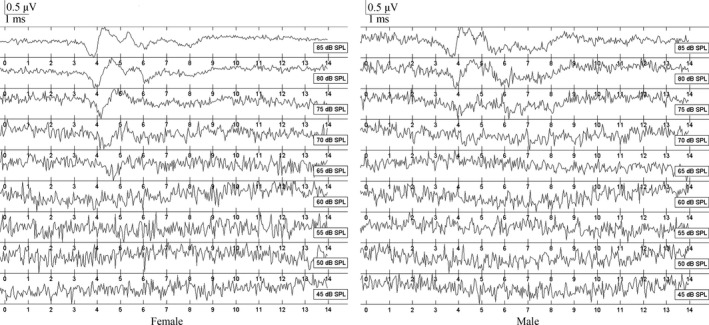
Auditory brainstem response (ABRs) as a function of stimulus intensity evoked by tone pips of 1 kHz from female and male *Amolops torrentis*, which exhibit thresholds of 65 and 70 dB SPL, respectively

Auditory brainstem response wave morphology was not different for female and male frogs. Figure 4 depicts a representative ABR level series for a male and a female evoked by 1‐kHz tone pip stimuli. As shown, the threshold is 70 dB for the male and 65 dB for the female. ABRs evoked by tone pip and click stimuli typically showed valley–peak waveforms, although occasionally peaks were not obvious in response to relatively low SPL stimuli. When several valley waveforms appeared in sequence, valley 1 was the lowest (relative to baseline) in many cases and was taken to represent the peak of the compound action potential of the auditory nerve (Figure 4).

**Figure 5:**
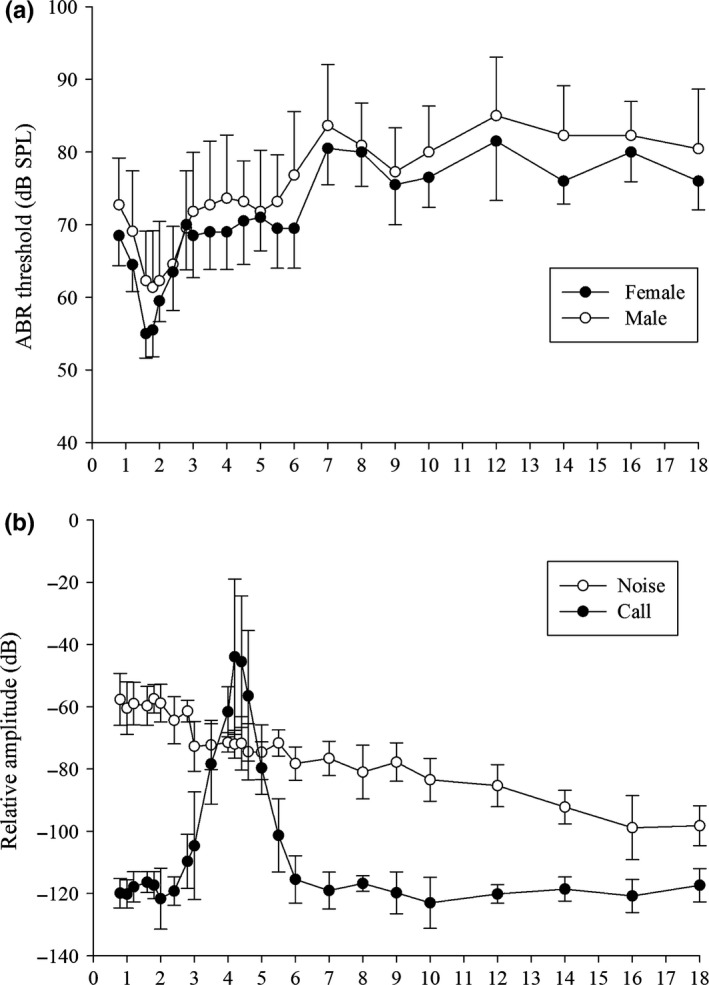
(a) Auditory brainstem response (ABR) mean thresholds (±*SD*) showing the best hearing sensitivity in the 1.6–2 kHz range (female: *n* = 10; male: *n* = 11). (b) Power spectra of advertisement calls and stream noise used in this study (±*SD*). The peak around 4,200 Hz represents the dominant frequency of the advertisement calls of *Amolops torrentis*

Auditory brainstem response audiograms for males and females for the 0.8–18 kHz range are shown in Figure 5. As shown in this figure, changes in male and female tone pip frequency thresholds across the entire frequency range were similar. A cross‐correlation analysis between male and female audiograms yielded a cross‐correlation coefficient with a median of −0.04 (95% CI: −0.36 to 0.85). For tone‐evoked ABRs, the repeated measures ANOVA revealed significant threshold differences in the 0.8–2.8 kHz (*F*_7,140_ = 28.812, *p *<* *.001) and 7–18 kHz (*F*_7,140_ = 4.093, *p* = .01) frequency ranges, but not in the 2.8–6 kHz (*F*_7,140_ = 1.209, *p* = .307) range. The sex (0.8–2.8 kHz: *F*_1,20_ = 3.269, *p* = .086; 2.8–6 kHz: *F*_1,20_ = 2.144, *p* = .160; 7–18 kHz: *F*_1,20_ = 3.890, *p* = .063) and frequency × sex (0.8–2.8 kHz: *F*_7,140_ = 2.039, *p* = .118; 2.8–6 kHz: *F*_7,140_ = 1.385, *p* = .227; *F*_7,140_ = 0.547; *p* = .655) interactions were also not significant across frequencies, although thresholds in females were noticeably lower compared with males (Figure 5). The region of the best hearing sensitivity of *A. torrentis* is centered around 1.8 kHz for both males and females, which is significantly lower than the DF of male advertisement calls (recordings *n* = 11, ABRs *n* = 21; Mann–Whitney rank sum test: *U* = 0, *p *<* *.001). The cross‐correlation analysis between the female auditory sensitivity curve and the spectra of male calls yielded cross‐correlation coefficients with a median of 0.08 (95% CI: −0.65 to 0.62), −0.06 (95% CI: −0.68 to 0.58), and 0.07 (95% CI: −0.29 to 0.25) at 0.8–2.8 kHz, 2.8–6 kHz, and 7–18 kHz, respectively.

### 3.3 Female phonotaxis

Females typically reached speakers in these experiments within 3–8 min. The phonotaxis results showed that low‐frequency calls with no extra noise were attractive to females compared to white noise (*n* = 23; Fisher's exact test: *p* = .038). When calls were broadcast antiphonally, however, females strongly preferred high‐frequency calls to low‐frequency calls in silent, low‐noise or high‐noise environments (Table 2).

**Table 2:** Responses of females to high‐frequency and low‐frequency calls in the phonotaxis tests

	Stimuli		Choices		*p*
	A	B	A	B	
No extra noise	High‐frequency	Low‐frequency	25	13	.011
Low noise	High‐frequency	Low‐frequency	19	9	.015
High noise	High‐frequency	Low‐frequency	19	7	.002

## 4 Discussion

Both the DF and SPL of the male calls of *A. torrentis* significantly exceed those of the background stream noise, indicating the male signals can be transmitted efficiently across the stream. Advertisement calls contained a series of 57 ± 13 notes with a mean DF of 4,318 Hz (Table 1), and the ratio of the maximum SPL of calls to that of the ambient background noise was 7.85. We compared these results with previous studies of two similar habitat species in Table 3 (Grafe et al., 2012; Preininger et al., 2013). Call differences in these streamside species suggest that *Micrixalus saxicola* and *Staurois* *parvus* improve the probability of sound signal recognition and detection by increasing the frequency of advertisement calls alone, while *A. torrentis* relies on increasing frequency, vocal amplitude, and call duration. These variations are examples of possible adaptations and/or phenotypic plasticity in noisy environments. Many birds and mammals increase the amplitude of vocalizations when exposed to increased noise levels (Lombard effect) (Brumm & Zollinger, 2011). The occurrence of this effect has been questioned in anurans; however, Halfwerk, Lea, Guerra, Page, and Ryan (2015) have recently found evidence showing that the Lombard effect may occur in anurans.

**Table 3:** Call characteristics of three streamside frog species

Call parameter	*Amolops torrentis*	*Micrixalus saxicola*	*Staurois parvus*
Dominant frequency (Hz)	4,318 ± 167	4,771 ± 29	5,578 ± 53
Notes per call	57 ± 13	21 ± 1	35 ± 3
Relative amplitude	80.3*/*62.4	69*/*67	62*/*72
Signal/noise	7.85	1.26	0.32

The ABR represents the output of synchronized neural activity in the auditory nerves and brainstem and has proven useful for determining auditory thresholds (Hall, 2007). In *A. torrentis*, ABR thresholds and auditory sensitivity tuning are not sexually dimorphic. This is similar to the condition in the gray tree frog (*Hyla chrysoscelis*) (Schrode et al., 2014; Zhang et al., 2012) and adds to growing evidence that auditory processing at the level of the auditory nerve is not dimorphic.

In many insects, fish, birds, and anurans, auditory sensitivity is closely matched with the spectral characteristics of conspecific vocalizations (Gall, Brierley, & Lucas, 2012; Ladich & Yan, 1998; Schmidt, Riede, & Römer, 2011; Simmons, 2013). The adaptive significance of such matching is that it increases the effectiveness of communication despite interference from many abiotic and biotic sources in the natural environment (Wiley & Richards, 1982). Furthermore, matching of auditory sensitivity and communication sound characteristics promotes speciation and diversification through sexual selection driven by sensory system specializations (Andersson, 1994; Boughman, 2002; Endler, 1992, 1993). Nevertheless, auditory tuning in female *A. torrentis* is substantially mismatched with the spectral characteristics of male acoustic signals. According to the sensory exploitation hypothesis, one component of a signal and receiver dyad can lag behind the other in the evolution of animal communication (Ryan, 1998). The present study suggests that stream noise promotes the evolution of higher DF call structure and that selection for higher frequency calls exerts great selective pressure on both males and females. Notably, females prefer the high‐frequency calls in phonotaxis experiments consistent with the idea that the preferences of the receiver coevolve with the characteristics of the sender. In contrast, the sensitivity of both the male and female auditory systems reflects the primitive condition due apparently to evolutionary conservation of the auditory periphery.

The occurrence of a mismatch between acoustic signals and hearing occurs in *Cyphoderris monstrosa*, because call signal characteristics exceed the coding capacity of the sensory system. Evidence from primary auditory receptor responses suggests that this auditory processing limitation may reflect the evolutionary origin of auditory frequency tuning from a generalized precursor (Mason et al., 1999). In mammals, birds, and reptiles, the mechanical organization of the cochlea plays a crucial role in determining the auditory frequency range (Ruggero & Temchin, 2002). Amphibians rely on two primary inner ear auditory organs, the amphibian papilla (AP) which controls low frequency sensitivity and the BP which controls high frequency sensitivity. Hearing in frogs is largely restricted by the responses of the auditory papillae at high frequencies where transmission losses occur due to the extracolumella–columella link (Narins et al., 2004). Moreover, frequency sensitivity may also be determined by electrical resonance or phenotypic plasticity. Additional morphological features are needed for high‐frequency auditory sensitivity in anurans. For example, the sunken tympana of males may support secondary resonant frequencies, which play a key role in high‐frequency hearing sensitivity in the Chinese concave‐eared frog (Feng et al., 2006). The substantial mismatch between auditory sensitivity and call structure in *A. torrentis* most likely is due to the retention of primitive peripheral auditory system morphological features derived from a common nonstreamside ancestor. Furthermore, studies on recognition space indicate that signal‐processing traits do not act as strictly matched filters when considering interactions between individuals within a complex assemblage (Amézquita et al., 2011). *A. torrentis* lacks such a complex ecological niche and the relevance of the recognition space of calls needs further study.

In this study, it is possible that a high‐frequency region of auditory sensitivity exists between 3 and 6 kHz in females insofar as the curve is relatively flat in this region in females, while males are not sensitive in this region (Figure 5). This difference may reflect sexual dimorphism due to a relevant difference in BP sensitivity, perhaps resulting from the different evolutionary pressures acting on males and females. In some species, female audiograms are characterized by two obvious regions of enhanced sensitivity which correspond to the AP and BP, respectively (Wilczynski et al., 2001). In view of the fact that the tuning curve of this second region is relatively flat in female *A. torrentis* and considering that male *A. torrentis* seem not to be sensitive in this region (Figure 5), it is possible that hearing sensitivity in this higher frequency region is still being acted on by selection and still evolving. This also suggests that the substantial mismatch discussed above existed during previous evolutionary stages.

According to the acoustic adaptation hypothesis, long‐term exposure to persistently high background noise levels, such as wideband river noise, might select for an upward shift in vocalization frequencies (Slabbekoorn & Peet, 2003). Low‐frequency calls would be masked by the fast‐flowing forest streams whose spectral energy is mainly below 4 kHz at the research site. Selection should therefore have favored the evolution of higher call frequencies in the ancestor of *A. torrentis* because such calls would be less likely to be masked by background noise. Females may originally have preferred low‐frequency vocalizations; however, low‐frequency calls would be more difficult to detect disrupting sexual selection.

It might be expected that females would prefer low‐frequency calls in the absence of noise and high‐frequency calls in the presence of noise, as hearing in *A. torrentis* is most sensitive at low frequencies. Yet females in the phonotaxis experiments preferred high‐frequency calls regardless of whether background noise similar to running water was added or not. These results suggest that preference for high‐frequency calls is not facultative, but is an adaptation which has coevolved with high‐frequency call production in males.

The perceptual basis for high‐frequency preference in *A. torrentis* cannot be due to peripheral sensory mechanisms because the results of the ABR experiments strongly favor the idea that hearing sensitivity is best in the low‐frequency range. Thus, it is likely that preference for high‐frequency male calls is based on central nervous system specializations. In many species, species‐specific signal processing adaptations involve the peripheral nervous system (Simmons, 2013). However, in a complex noisy environment, the production of communication sounds outside the sensitivity range of the filter would still be adaptive if these signals avoid the masking effect of environmental sounds (Capranica & Moffat, 1983; Wehner, 1987). In the present study, therefore, acoustic communication in *A. torrentis* may reflect two kinds of evolutionary adaptation: changes in the energy distribution of male calls to higher frequencies and changes in the central nervous system shifting female preferences to high‐frequency calls.

In summary, our results support the idea that hearing in *A. torrentis* is an exception to the matched filter hypothesis. In this species, female auditory tuning is not well matched with the spectral characteristics of male acoustic signals. Females have thus evolved a stable preference for high‐frequency male calls under long‐term stream noise interference, which matches the energy distribution of male advertisement calls, despite the fact that the sensitivity range of the auditory filter remains centered at low frequencies. The present study highlights the important role of central auditory processes in solving the problem of sound communication in noisy environments. Future studies are needed to determine whether this is a unique adaptation in this species or a more common evolutionary adaption in streamside species.

## Conflict of Interest

None declared.
